# Detection of Changes in Monoamine Neurotransmitters by the Neonicotinoid Pesticide Imidacloprid Using Mass Spectrometry

**DOI:** 10.3390/toxics10110696

**Published:** 2022-11-17

**Authors:** Anri Hirai, Ryo Yamazaki, Atsushi Kobayashi, Takashi Kimura, Kei Nomiyama, Shuichi Shimma, Shouta M. M. Nakayama, Mayumi Ishizuka, Yoshinori Ikenaka

**Affiliations:** 1 Laboratory of Toxicology, Department of Environmental Veterinary Sciences, Faculty of Veterinary Medicine, Hokkaido University, Sapporo 060-0818, Japan; 2 Laboratory of Comparative Pathology, Department of Veterinary Clinical Medicine, Faculty of Veterinary Medicine, Hokkaido University, Sapporo 060-0818, Japan; 3 Center for Marine Environmental Studies, Ehime University, Matsuyama 790-8577, Japan; 4 Department of Biotechnology, Graduate School of Engineering, Osaka University, 2-1 Yamadaoka, Suita 565-0871, Japan; 5 Translational Research Unit, Veterinary Teaching Hospital, Faculty of Veterinary Medicine, Hokkaido University, Sapporo 060-0818, Japan; 6 One Health Research Center, Hokkaido University, Sapporo 060-0818, Japan; 7 Water Research Group, Unit for Environmental Sciences and Management, North-West University, 11 Hoffman Street, Potchefstroom 2531, South Africa

**Keywords:** monoamine neurotransmitters, mass spectrometry, neonicotinoid, imidacloprid, open field test, neurotoxicity

## Abstract

Monoamine neurotransmitters (MAs), including dopamine (DA) and serotonin (5-HT), regulate brain functions such as behavior, memory, and learning. Neonicotinoids are pesticides that are being used more frequently. Neonicotinoid exposure has been observed to produce neurological symptoms, such as altered spontaneous movements and anxiety-like behaviors, which are suspected to be caused by altered MA levels. However, current neurotoxicity tests are not sufficiently sensitive enough to make these determinations. In this study, we performed some behavior tests, and derivatization reagents to improve the ionization efficiency, which was applied to liquid chromatography mass spectrometry (LC-MS/MS) to reveal the effect of neonicotinoid administration on MAs in the brain. We orally administered the neonicotinoid imidacloprid (0, 10, and 50 mg/kg body weight) to C57BL/6NCrSlc mice. In the behavior tests, a decrease in activity was observed. The LC-MS/MS quantification of MAs in various brain regions showed a decrease in some MA levels in the olfactory bulb and the striatum. These results showed, for the first time, that even a low dose of imidacloprid could alter MA levels in various parts of the brain.

## 1. Introduction

Neonicotinoid insecticides (NNs) are pesticides whose use has rapidly expanded since their registration in 1992 [1,2]. NNs have a similar structure to nicotine and target the insect nicotinic acetylcholine receptor (nAChR). nAChRs are abundantly expressed in the central nervous system of insects, and when NNs bind to them, the excitation is sustained, resulting in insecticidal effects [3,4]. Vertebrates, including mammals, also have nAChRs, but the different structures of these receptors are believed to result in lower toxicity by NNs [5,6,7,8]. NNs have thus become one of the most used pesticides in the world because of their high safety for mammals and in a wide range of applications. However, NN-induced toxicity has still been reported in recent years, including neurotoxicity, reproductive toxicity, and hepatotoxicity when administered to mice and rats [5,6,7,8,9,10,11,12,13,14,15]. In particular, there have been many reports of neurotoxicity, including increased anxiety-like behavior and changes in spontaneous locomotion [9,16,17,18,19,20,21], and Hirano et al. [18] ) found that clothianidin (CLO), an NN, increased anxiety-like behavior even at doses below the no observed adverse effect level (NOAEL). Sex differences in these behavioral effects have also been reported, suggesting that males are more susceptible to these effects than females [22]. The detailed mechanism of such neurotoxicity is still unknown.

“Monoamine neurotransmitters” (monoamines) is a general term for neurotransmitters with a single amino group, and they are known to regulate brain functions such as behavior, memory, emotion, and learning. For example, the monoamine DA is known to be involved in motor control and learning, while serotonin is known to be involved in anxiety and memory [15]. Thus, monoamines play an important role in the regulation of brain function, and abnormalities in their functioning are thought to be involved in the development of neurological disorders such as depression, anxiety disorders, and Parkinson’s disease. Although there are many causes of monoamine abnormalities, pesticides that target the central nervous system are being considered as one of the causes [23]. Some chemicals, such as NN, exert their toxic effects by acting on receptors in the central nervous system, disrupting neurotransmitters such as monoamines. For example, Oliveira et al. [14] reported that intracerebral administration of CLO and thiamethoxam (TMX) enhanced dopamine (DA) release in the striatum, and these change in monoamines in the brain are suspected to be responsible for the NNs-induced behavioral changes. However, few studies have investigated the effects of NN on monoamines, and thus, insufficient knowledge currently exists. The change in neurotransmitters causes detrimental effects on the body, most of which manifest as abnormalities in higher brain functions, such as behavior, memory, and learning, without histopathological changes [24]. However, current neurotoxicity studies focus on symptom observation, behavioral tests, and histopathological examinations after the administration of test substances to evaluate toxicity, making it difficult to detect neurodisruptions such as monoamine abnormalities. It is essential to use a simple, highly sensitive, and quantitative detection method for more accurate toxicity assessment [25].

Liquid chromatography triple-quadrupole mass spectrometry (LC-MS/MS) is a detection method for measuring substances in a highly sensitive and quantitative manner by combining mass spectrometry, which ionizes substances in a sample and measures the mass/charge ratio specific to each substance, with high-performance liquid chromatography. This method allows for the detection of neural alterations, including abnormalities in monoamines, which cannot be detected by conventional methods. 

**Figure 1:**
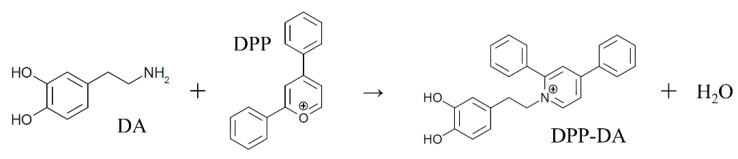
Chemical reaction of DPP-derivatization. DA, Dopamine; DPP, Tetrafluoroborate salt of 2,4-diphenyl-pyranylium.

There have been several previous studies on the detection of monoamines using LC-MS/MS [26,27,28,29]. However, the octadecylsilyl columns used for liquid chromatography of monoamines have weak retention, resulting in low ionization efficiency and difficulty in separating impurities. Therefore, the detection sensitivity of this technology is not high. Since neurotransmitters, including monoamines, exert their effects in minute amounts, more sensitive analytical methods are required to detect their fluctuations. It has been reported that the use of tetrafluoroborate salt of 2,4-diphenyl-pyranylium (DPP), a monoamine derivatization reagent, increases the ionization efficiency and enables highly sensitive monoamine detection [30,31,32,33]. Thus, the localization of a wide variety of molecular species can be visualized by performing mass spectrometry on tissue sections. DPP is part of the pyrilium group and reacts with the amino group of monoamines (Figure 1). Although there are no examples of LC-MS/MS applications, this reagent has the potential to be used for highly sensitive analyses of monoamines in LC-MS/MS. Therefore, the main objective of this study was to investigate the effect of NN on monoamine production in the brain by applying DPP to LC-MS/MS analysis.

## 2. Materials and Methods

All experiments were conducted in accordance with ARRIVE guidelines.

### 2.1. Chemicals

Imidacloprid (IMI) was purchased from Kanto Chemical Co., Inc. (Tokyo, Japan). DA, 3-methoxytyramine HCl (3-MT), 3-methoxytyramine-d4 HCl, and serotonin were purchased from Sigma-Aldrich (St. Louis, MO, USA). Serotonin-d4 hydrochloride and histamine were purchased from Toronto Research Chemicals, Inc. (Toronto, ON, Canada), and dopamine-d4 and histamine-d4 were purchased from WATARAI Co., Ltd. (Kanagawa, Japan). DPP, which was originally purchased from Sigma Aldrich (St. Louis, MO, USA), was donated by Associate Professor Shuuichi Shimma, Department of Biotechnology, Graduate School of Engineering, Osaka University. Corn oil and formic acid were purchased from Wako Pure Chemicals Co., Inc. (Tokyo, Japan); dimethyl sulfoxide (DMSO) was purchased from Nacalai Tesque, Inc. (Kyoto, Japan); triethylamine (TEA) was purchased from Tokyo Chemical Industry Co., Ltd. (Tokyo, Japan); and methanol (MeOH) was purchased from Kanto Chemical Co., Inc.

### 2.2. Animals

All of the animal experiments were approved by the Experimental Committee of the Faculty of Veterinary Medicine, Hokkaido University. All of the animal experiments were conducted in accordance with the Guide for the Care and Use of Laboratory Animals and with the Association for the Assessment and Accreditation of Laboratory Animal Care International (AAALAC; approval number: 18-0061). Male C57BL/6NCrSlc mice aged–10–12 weeks were obtained from Sankyo Labo Service Co., Inc. (Tokyo, Japan) and kept in a 12-h light/dark cycle (7:00 a.m. to 7:00 p.m.: light, 7:00 p.m. to 7:00 a.m.: dark) at a room temperature of 22 ± 1 °C and humidity of 70 ± 5%. Food (breeding solid feed for mice, rats, and hamsters: CE-2, CLEA Japan, Inc., Tokyo, Japan) and tap water were provided to the mice ad libitum. The food and water were changed twice a week, and the cages were changed once a week. The experimental procedures were conducted after an acclimation period of at least two weeks.

### 2.3. IMI Administration, Euthanasia, Dissection, and Organ Collection

#### 2.3.1. For the Behavioral Test

The mice (*n =* 48) were randomly divided into two test groups, one for the elevated plus maze test (EPM) (*n =* 24) and one for the open field test (OF) (*n =* 24). Within each test group, there were three treatment groups (*n =* 8/group, following previous study [34]): control, low-dose, and high-dose groups. IMI dissolved in 2.5% (*v*/*v*) DMSO in corn oil was orally administered to the low-dose group at 10 mg/kg body weight and to the high-dose group at 50 mg/kg body weight. The control group received 2.5% (*v*/*v*) DMSO in corn oil orally at a dose of 10 mL/kg body weight. Oral administration with sonde (FUCHIGAMI Co., Ltd., Kyoto, Japan) was performed under mild isoflurane anesthesia. Afterward, the mice were placed in individual cages in a dark room. Behavioral tests were then conducted 1 h after oral dosing. All IMI administrations and behavioral tests were performed during the light cycle of the housing facility. The dose of IMI used in this experiment was set at 10 mg/kg (low-dose group), which is the NOAEL, and approximately 46 mg/kg (high-dose group), which is the lowest observed adverse effect level (LOAEL) [35].

#### 2.3.2. For the Monoamine Concentration Measurement

The mice (*n =* 24) were randomly divided into three treatment groups (*n =* 8/group): control, low-dose, and high-dose groups. Each group received 2.5% (*v*/*v*) DMSO in corn oil or IMI dissolved in 2.5% (*v*/*v*) DMSO in corn oil, as described above. One hour after administration, cardiac blood sampling was performed under isoflurane anesthesia, followed by euthanasia by cervical dislocation.

The brain was sectioned using a brain matrix (stainless steel, mouse (40–75 g) Coronal, CellPoint, product code: 15831110, model: 69-2175-1, Bio Research Center Co., Ltd., Nagoya, Japan) in coronal sections containing the striatum (Bregma 1.00 mm–0.00 mm) and a coronal section containing the hippocampus (Bregma −3.00–−4.00 mm). The sections were visually checked and then sampled by punching out tissue using a disposable biopsy punch (φ2 mm, Lot No. 19L48, Kai Industries Co., Ltd., Tokyo, Japan). The hippocampus was separated from the abovementioned section using tweezers and then sampled. Other regions (cerebral cortex, brainstem, cerebellum, and olfactory bulb) were also isolated and sampled. Each brain region was weighed (shown in Table 1), placed in 1.5 mL tubes (olfactory bulb, striatum, and hippocampus) or 2.0 mL tubes (cerebral cortex, brainstem, and cerebellum), flash frozen in liquid nitrogen, and stored at −80 °C. Blood obtained by cardiac puncture using the needle filled with heparin was kept on ice and then centrifuged (4 °C, 4000× *g*, 10 min). The plasma was stored at −80 °C. The brain and plasma samples were used to measure monoamine levels.

### 2.4. Behavioral Test

#### 2.4.1. Open Field Test (OF)

OF is a test system that evaluates spontaneous locomotion, activity, and anxiety levels using exploratory behavior and responsiveness in novel environments. The OF was performed using a square open field box for mice (model LE802S; Panlab, Bio Research Center Co., Ltd., Nagoya, Japan). The apparatus consisted of a 45 cm × 45 cm square floor surface and 40 cm walls. The central 22.5 cm × 22.5 cm floor of the apparatus was set as the center area and the remaining floor as the side areas. The brightness was set such that the center of the device was approximately 20 lx.

One mouse was placed in the center of the floor of the apparatus with its head facing the wall opposite the experimenter and allowed to freely explore the apparatus for 5 min. We were aware of the group allocation, but their behavior was objectively analyzed using SMARTV3.0 (Serial No. DCF76-90C; Panlab, Bio Research Center Co., Ltd.). The distance traveled, number of area movements, average movement speed, and resting time were used to measure spontaneous locomotion and activity, and the time spent in the center area was used as an index of anxiety-like behavior. The average movement speed was the average movement speed during the activity time (5 min minus resting time).

#### 2.4.2. Elevated Plus Maze Test (EPM)

EPM is a behavior test used to evaluate anxiety behavior. The EPM was performed using an elevated plus maze for mice (model LE842A; Panlab, Bio Research Center Co., Ltd.). The apparatus consisted of an aisle with walls (closed arm) and an aisle without walls (open arm) crisscrossing each other at a height of 40 cm above the floor. The length of each passage was 29.5 cm, the width was 6 cm, and the height of the closed-arm wall was 15 cm. The brightness was set such that the center of the device was 20 lx.

One mouse was placed in the center of the apparatus with its head facing the open arm side opposite the experimenter and allowed to freely explore the apparatus for 5 min as in OF test. SMARTV3.0 (Serial No. DCF76-90C; Panlab, Bio Research Center Co., Ltd.) was used to analyze the behavior, and the ratio of the time spent in the open arm to the measurement time and the ratio of the number of intrusions into the open arm to the number of intrusions into both arms were used as indicators of anxiety-like behavior.

### 2.5. Investigation of the Conditions for DPP Derivatization and Analysis

#### 2.5.1. Target Substances

Among the monoamine neurotransmitters, there were seven to be measured: DA, 3-methoxytyramine, l-DOPA, norepinephrine, normetanephrine, serotonin (tryptophan metabolite), and histamine (histidine metabolite), all belonging to the tyrosine metabolism system. However, l-DOPA, norepinephrine, and normetanephrine were poorly derivatized by DPP, making accurate quantification difficult. Therefore, the final four substances to be measured were DA, 3-methoxytyramine, serotonin, and histamine.

#### 2.5.2. LC/MS Measurement Conditions

An Agilent 1290 Infinity UHPLC system (Agilent Technologies, Tokyo, Japan) was used for LC, and an Agilent 6495 triple quadrupole mass spectrometer (Agilent Technologies) was used for MS. A 100 mm × 3.0 mm InertSustain AQ-C18 column with a particle size of 1.9 μm (GL Science, Tokyo, Japan) was used, and the temperature was set to 60 °C. Distilled water (DW) containing 0.1% formic acid was used for mobile phase A, and acetonitrile containing 0.1% formic acid was used for mobile phase B. The analytes were separated at a flow rate of 0.8 mL/min. The gradient was started at 10% B, increased linearly to 95% B from 0.5 min to 4 min, maintained at 95% B for 1 min, and then returned to 10% B and equilibrated for 0.5 min before the next injection. The injection volume was 20 μL, and electrospray ionization (ESI) was performed in positive mode. The retention time (RT), multiple reaction monitoring (MRM) transition, and collision energy (CE) for each analyte are listed in Table 2.

#### 2.5.3. Investigation of Derivatization Reaction Methods

##### Preparation of Standard (STD) Mix and Internal Standard (IS) Mix

Each standard solution was mixed and diluted with 70% MeOH containing 0.1% formic acid to prepare a 1 ppm standard solution (hereafter, called STD mix). The same procedure was followed for the IS to obtain a 1 ppm internal standard solution (hereafter, called IS mix).

##### Preparation of the DPP Mix

The preparation of DPP solution was based on the method of Shimma et al. [32]. DPP was adjusted to 10 mg/mL with methanol and made into a DPP solution. As a dilution solvent, 8.1 mL of methanol, 5.4 mL of DW, and 9 µL of TEA were mixed. The derivatization reagent was a solution made by mixing the resulting diluted solvent and DPP solution to a composition of 69:6 DPP mix.

##### Investigation of Derivatization Reaction Time

Ten microliters each of the DPP mix and STD mix were combined and derivatized at 60 °C. Samples were collected after 1, 2, 3, 4, 5, and 6 h, diluted with 80 µL of distilled water, and measured by LC-MS/MS.

##### Investigation of Derivatization Reaction Temperature

Ten microliters each of the DPP mix and STD mix were combined and derivatized at room temperature (about 25 °C), 40 °C, or 60 °C. Samples were collected after 1, 2, 3, 4, 5, and 6 h, diluted with 80 µL of distilled water, and measured by LC-MS/MS.

##### Calculation of Instrument Detection Limit (IDL) and Method Detection Limit (MDL)

The IDL was calculated by repeating the measurement seven times with a standard solution corresponding to the lowest concentration (4 ppt) of the standard in the calibration curve. The standard deviation was then calculated from the obtained analytical value using the formula below.

To calculate the MDL, IS mix was added to mouse brain extract and diluted with MeOH until near the limit of quantification. After the diluted solution was derivatized with DPP, it was analyzed by the prescribed operation, and the obtained analytical value was converted to sample concentration. This operation was repeated seven times, and the MDL was calculated from the standard deviation at that time, using the formula below:

IDL = 2 × s × 1.943,(1)

MDL = 2 × s × 1.943,(2)

where s is the standard deviation and 1.943 is t (6, 0.05) (i.e., the Student’s t-value with six degrees of freedom, risk rate 5% (one-sided)). The IDL and analytical MDL are listed in Table 3. In addition, the accuracy and precision of this analytical method are listed in the Supplementary Materials (Table S1).

### 2.6. Measurement of Monoamine Concentration

#### 2.6.1. Measurement of Monoamine Concentration in Brain Tissue

We used the brains that had been collected in Section 2.3.2. First, 200 ppb, 20 µL (for striatum); 10 ppb, 20 µL (for hippocampus); or 100 ppb, 20 µL (for olfactory bulb, cerebral cortex, brainstem, and cerebellum) of IS mix (1 ppm in 70% MeOH containing 0.1% formic acid; diluted with MeOH as appropriate) was added as an internal standard to 1.5 mL tubes (striatum, hippocampus, olfactory bulb) and 2.0 mL tubes (cerebral cortex, brainstem, cerebellum) containing each brain region. Two zirconia beads (2.0 mm, Lot No. 0894-T40, Toray Industries, Inc., Tokyo, Japan) were placed in each 1.5 mL tube, and one zirconia bead (5.0 mm, ZZ50-0001, Bio-medical Science Co, Ltd., Tokyo, Japan) was placed in each 2.0 mL tube. Ice-cold 0.05% acetonitrile formate was added (200 µL for striatum and hippocampus, 100 µL for olfactory bulb, 300 µL for cortex and brainstem, 400 µL for cerebellum) and homogenized in a Tissue Lyser (1 min, 30/s; Retsch, QIAGEN K.K., Tokyo, Japan). Then, each tube was centrifuged (10,000× *g*, 10 min, 25 °C). Ten microliters of the supernatant and 10 µL of the DPP mix were added to 8 tubes, and the derivatization reaction was carried out in a thermal cycler at 60 °C for 4 h. After the completion of the reaction, 80 µL of distilled water was added to each tube for dilution, and the target substances were measured by LC-MS/MS. Target monoamines referred in 2.5.1. were quantified using an internal standard method. Each peak shape was checked for the analysis; a peak with a signal-to-noise ratio > 10 was adopted as the peak. Other than that, it was marked as “N.D. (not detected)”.

**Figure 2:**
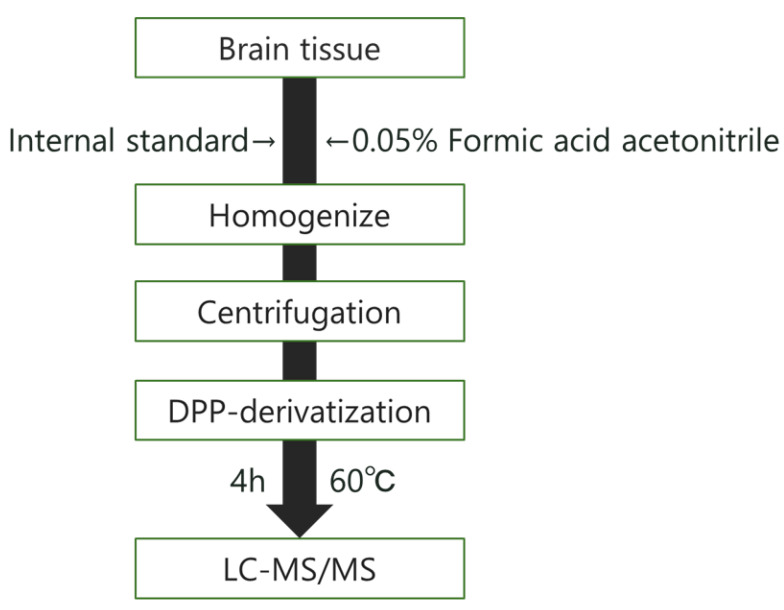
Analytical procedure of DPP-derivatization. DPP, Tetrafluoroborate salt of 2,4-diphenyl-pyranylium; LC-MS/MS, Liquid chromatography-tandem mass spectrometry.

The analysis method that was established based on the above findings is shown in Figure 2.

#### 2.6.2. Measurement of Plasma Monoamine Concentration

The plasma stored at −80 °C was thawed, 50 µL was sampled in a 1.5 mL tube, and 100 ppb (20 µL) of IS mix was added. Fifty microliters of ice-cold 0.05% formic acid acetonitrile was then added, and the mixture was centrifuged (10,000× *g*, 10 min, 25 °C). Ten microliters of the supernatant and ten microliters of the DPP mix were added to eight tubes, and the derivatization reaction was carried out in a thermal cycler at 60 °C for 4 h. After completion of the reaction, 80 µL of distilled water was added to each tube, and the target substance were measured by LC-MS/MS. Each data was checked similarly to 2.6.1.

### 2.7. Statistical Analysis

Statistical analysis was performed using Excel (2019) and JMP® (SAS Institute Inc., Cary, NC, USA). Analysis of variance was performed with the Bartlett test (*p* < 0.025). The Tukey-HSD test was used for comparison of reaction times, and the Dunnett test was used for comparison between groups in the results of behavioral tests and measurements of monoamine concentrations. Data are presented as mean ± standard error, and the criterion for significant difference was *p* < 0.05.

## 3. Results

### 3.1. Investigation of Conditions for DPP Derivatization Used in the Analysis Method

**Figure 3:**
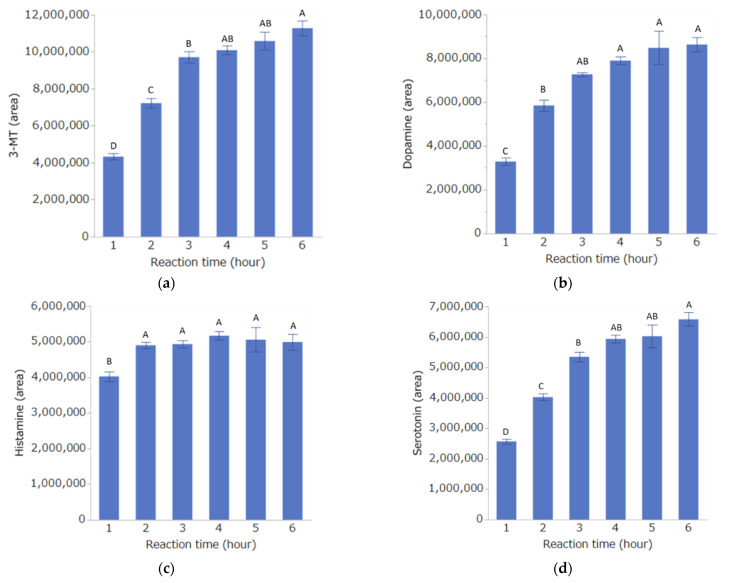
Effect of reaction time on derivatization with DPP. Detection of 3-MT (**a**), dopamine (**b**), histamine (**c**), and serotonin (**d**) by LC-MS/MS. Bar graphs indicate the mean ± SE of each condition (*n =* 5 per condition). Different characters indicate significant differences, while identical characters indicate non-significant differences, as determined by Tukey-HSD tests.

The results of the reaction time study are shown in Figure 3. For each substance, the concentration (area value) increased in a time-dependent manner. For all of the substances except histamine, the highest concentration was obtained 6 h after the start of the reaction, but there was no significant difference between this time and 4 h. Thus, the reaction time was set to 4 h.

**Figure 4:**
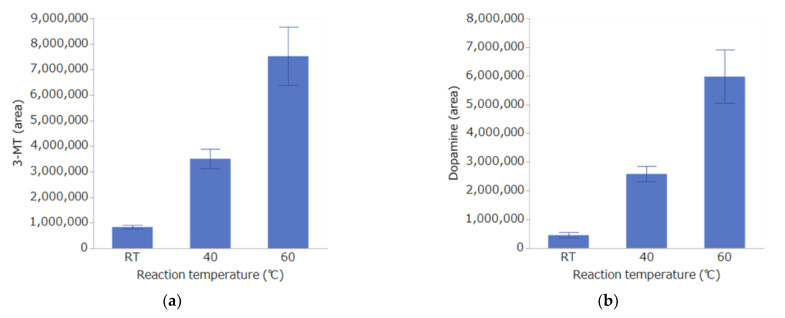

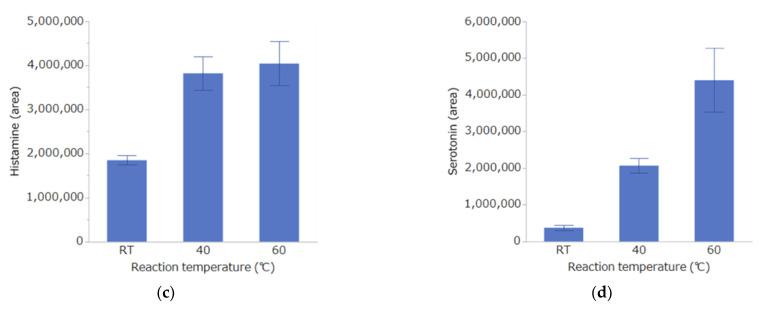
Effect of reaction time on derivatization with DPP. Detection of 3-MT (**a**), dopamine (**b**), histamine (**c**), and serotonin (**d**) by LC-MS/MS. Bar graphs indicate the mean ± SE of each condition (*n =* 5 per condition).

The results of the reaction temperature study are shown in Figure 4. The concentration increased in a temperature-dependent manner, and the reaction efficiency was maximal at 60 °C for all of the substances. Therefore, the reaction temperature was set to 60 °C. 

### 3.2. Open Field Test

**Figure 5:**
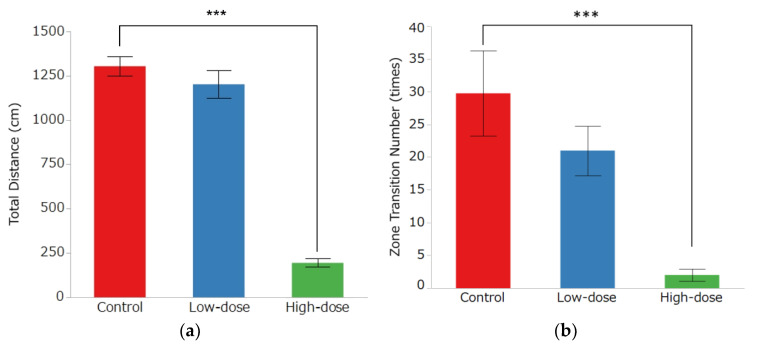

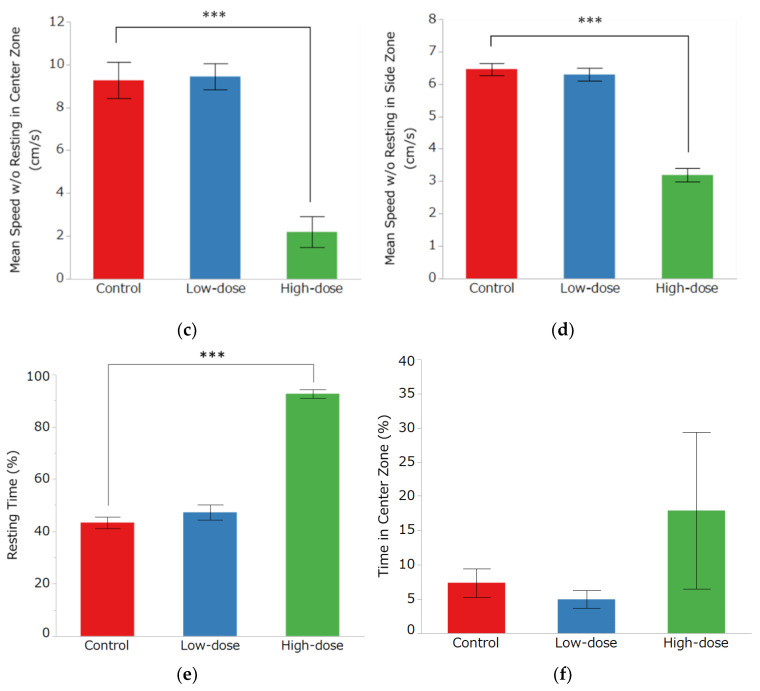
Behavioral effects of IMI administration in the open field test. (**a**) Total distance, (**b**) zone transition number, and mean speed without resting in (**c**) center zone and (**d**) side zone were significantly lower in the high-dose group than in the control group. (**e**) Resting time was significantly higher in the high-dose group than in the control group. (**f**) There were no significant differences among all groups in the time in center zone. Bar graphs indicate the mean ± SE of each group (*n =* 8 per group), *** *p* < 0.001, Dunnett’s test.

The OF results are shown in Figure 5. In the OF group, there was a significant decrease in the distance traveled from 1304 ± 55 cm in the control group and 1202 ± 77 cm in the low-dose group to 195 ± 24 cm in the high-dose group (Figure 5a, *p* < 0.0001). The number of area movements was 30 ± 7 times in the control group, 21 ± 4 times in the low-dose group, and 2 ± 1 times in the high-dose group, with a significant decrease in the high-dose group (Figure 5b, *p* = 0.0004). In the center area, the mean movement speed was 9.27 ± 0.86 cm/s in the control group, 9.45 ± 0.61 cm/s in the low-dose group, and 2.19 ± 0.72 cm/s in the high-dose group. In the side area, the mean movement speed was 6.46 ± 0.19 cm/s in the control group, 6.29 ± 0.20 cm/s in the low-dose group, and 3.20 ± 0.20 cm/s in the high-dose group; in both areas, there was a significant decrease in mean movement speed in the high-dose group (Figure 5c,d, *p* < 0.0001). The resting time was 43.4 ± 2.22% in the control group, 47.30 ± 2.93% in the low-dose group, and 92.67 ± 1.60% in the high-dose group, with a significant increase in the high-dose group (Figure 5e, *p* < 0.0001). Although the average time spent in the center area was higher in the high-dose group (17.91 ± 11.45%) than in the control group (7.38 ± 2.09%) and low-dose group (4.96 ± 1.31%), the differences were not statistically significant (Figure 5f). 

### 3.3. Elevated Plus Maze Test

**Figure 6:**
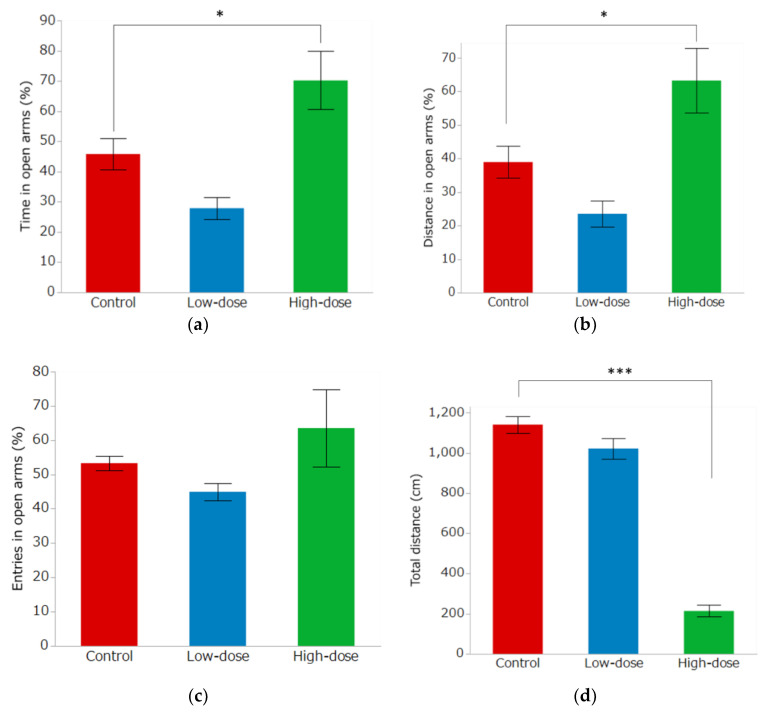
Behavioral effects of IMI administration in the elevated plus maze test. The percentage of time (**a**) and distance (**b**) spent in the open arm were significantly higher in the high-dose group than in the control group. (**c**) There were no significant differences in the percentage of entries in the open arm. (**d**) The total distance traveled was significantly decreased in the high-dose group. Bar graphs indicate the mean ± SE of each group. (*n =* 8/group), * *p* < 0.05, Dunnett’s test; *** *p* < 0.001, Dunnett’s test.

The EPM results are shown in Figure 6. The percentage of time spent in the open arm was 45.80 ± 5.24% in the control group, 27.86 ± 3.68% in the low-dose group, and 70.20 ± 9.60% in the high-dose group, and the percentage of distance spent in open arms was 38.95 ± 4.76%, 23.56 ± 3.85% and 63.25 ± 9.62%, in the control group, low-dose group, high-dose group, respectively. For these parameters, there was a significant increase in the high-dose group compared to the control group (Figure 6a,b, *p* < 0.05). In contrast, the percentages of open arm entries were 53.27 ± 2.09% in the control group, 44.93 ± 2.48% in the low-dose group, and 63.52 ± 11.31% in the high-dose group, which were not significantly different (Figure 6c). The total distance traveled in the control, low-dose and high-dose group was 1140.7 ± 41.08 cm, 1021.7 ± 51.91 cm and 214.1 ± 28.49 cm, respectively, was significantly less in the high-dose group than in the control group (Figure 6d, *p* < 0.0001).

### 3.4. Measurement of Monoamine Concentrations in Various Parts of the Brain

Using the analytical method established in this study, we measured monoamine concentrations in various regions of the brain. The measurement sites were the striatum, hippocampus, cerebral cortex, cerebellum, brain stem and olfactory bulb, and the results were compared between the control and exposed groups (Table 4). In the cerebellum, 3-MT was significantly elevated in the high-dose group (*p* = 0.0159), while it was significantly decreased in the olfactory bulb in the high-dose group (*p* = 0.0011). DA was significantly reduced in the cerebellum in the high-dose group (*p* = 0.0007), and there was a significant dose-dependent reduction in DA in the olfactory bulb (low-dose group, *p* = 0.0061; high-dose group, *p* = 0.0004). In addition, a dose-dependent decreasing trend was observed in the striatum, although there was no significant difference between groups. Histamine levels were significantly decreased in the striatum in both the low-and high-dose groups (*p* < 0.0001). Significant decreases were also observed in the cerebellum (high-dose group) and olfactory bulb (low-dose and high-dose groups) (cerebellum, high-dose group: *p* = 0.013; olfactory bulb, low-dose group: *p* = 0.0271, high-dose group: *p* = 0.037). Serotonin was significantly reduced in the striatum, from 438.0 ± 44.8 ng/g in the control group to 193.3 ± 33.7 ng/g in the low-dose group and 320.2 ± 14.8 ng/g in the high-dose group (low-dose group, *p* < 0.0001; high-dose group, *p* = 0.0391, compared to the control group). At the other sites, no significant difference was observed for any of the monoamines.

### 3.5. Measurement of Plasma Monoamine Concentration

The results of the measurements of monoamine concentrations in the plasma are shown in Table 5. Histamine was reduced in a dose-dependent manner in the IMI: 131 ± 32.78 ng/mL in the control group, 58.90 ± 6.88 ng/mL in the low-dose group, and 45.08 ± 3.43 ng/mL in the high-dose group (low-dose group, *p* = 0.03; high-dose group, *p* = 0.0099). There were no significant changes in 3-MT and serotonin levels, and DA was not detected.

## 4. Discussion

NNs such as IMI are known to exhibit selective toxicity to many pests with low toxicity to mammals. However, in recent years, mammalian toxicity, especially the effects on affective cognitive behavior, has been reported. Hirano et al. [18] administered a single oral dose of CLO, a type of NN, to mice and performed EPM. As a result, the anxiety levels of the mice increased in a dose-dependent manner. The same investigators found that anxiety-like behavior was also increased by CLO below the NOAEL. Yoneda et al. [19] also reported that long-term administration of dinotefuran to mice caused a dose-dependent increase in spontaneous locomotor activity. Chronic administration of IMI has been reported to cause a decrease in voluntary locomotion, an increase in resting time, and depression [9,16,21]; since monoamines regulate brain functions such as behavior, memory, emotion, and learning, monoamine disruption may be involved in the development of those symptoms.

In this study, we first confirmed the effects of acute exposure to IMI on affective and cognitive behaviors using OF and EPM in mice, assessing exploratory behavior and responsiveness to novel environments as indicators of spontaneous locomotion, activity, and anxiety levels. Normally, animals exposed to a novel environment avoid the center of the device (center area) and perform exploratory behavior near the wall. In contrast, animals which are administered benzodiazepine anxiolytic drugs increase their exploratory behavior in the center area [36]. These effects are thought to be a state representing release of behavioral inhibition that is based on anxiety and fear in a novel environment [37]. However, it is difficult to conclusively evaluate anxiety levels using this test system alone, and it is important to include other test systems. In the present study, the distance traveled, number of area movements, average speed of movement, and resting time were used to measure spontaneous locomotion and activity, while the time spent in the center area was used as an index of anxiety-like behavior. As a result, the distance traveled, number of area movements, and average speed of movement were significantly reduced in the high IMI-dose group. In contrast, the resting time was markedly increased in the high-dose group. The time spent in the center area did not significantly change in either group. These results indicate that the administration of high-dose IMI caused a decrease in spontaneous locomotion and activity. These results are consistent with those of the previous studies. In contrast, no anxiety-like behaviors were observed. In the EPM, we assessed anxiety by taking advantage of the fact that mice prefer the wall side of an enclosure and avoid heights [38]. On the open arm, mice feel fear and anxiety due to exposure to heights; thus, we can evaluate their anxiety levels by measuring the time and distance spent on the open arm or the number of intrusions. In this study, there was no significant change in the entries into the open arm between the control and exposed groups, but the time and distance spent in the open arm increased in the high-dose group. However, because the total distance traveled was significantly less in the high-dose group, these results did not reflect a decrease in anxiety levels, but rather a decrease in activity and an increase in rest time, which might have increased the time spent in the open arms.

Since the results of the behavioral tests showed that IMI caused a decrease in activity, the next step in this study was to measure monoamines in the brains of IMI-exposed mice using LC-MS/MS. Prior to these measurements, in this study, we investigated the appropriate LC-MS/MS analytical conditions for the determination of monoamine neurotransmitters using the derivatizing reagent DPP. DPP specifically reacts with monoamines, and its use in MSI has been reported [30,31,32,33]. DPP derivatization for MSI requires a reaction time of at least 24 h at room temperature [33,39], but a shorter reaction time is essential to establish a simpler analytical method for LC-MS/MS. Therefore, we investigated the optimal reaction time and temperature and found them to be 60 °C and 4 h. The extraction process before derivatization utilized the method of Maeda et al. [40], with modifications, to establish an analytical method for measuring monoamines via DPP derivatization. The limits of detection (LODs) of conventional LC-MS/MS methods for monoamine analysis in brain tissue were reported as 125 ppt to 3 ppb for 3-MT, 114 ppt to 4 ppb for DA, and 132 ppt to 44 ppb for serotonin [26,29,41,42,43,44]. However, the detection of histamine in brain tissue by LC-MS/MS has not been reported. In addition, these methods require pretreatment such as ion-exchange solid-phase extraction and drying under nitrogen gas to achieve highly sensitive measurements [29,41,42]. The LOD (MDL) of the analytical method established in this study was 2.08 ppt for 3-MT, 24.9 ppt for DA, 9.82 ppt for histamine, and 1.51 ppt for serotonin, achieving much higher detection sensitivity than conventional methods. We were able to simplify the pretreatment process, as we only needed to derivatize the extracts after brain tissue homogenization. In this study, only four monoamines were measured, but it is believed that the simultaneous detection of more monoamines could be realized in the future.

The measurement of monoamines in each brain region of the IMI-exposed mice by DPP derivatization LC-MS/MS showed that DA was significantly decreased in the cerebellum and olfactory bulb, and serotonin and histamine were significantly decreased in the striatum. DA is a neurotransmitter involved in motor coordination, feelings of pleasure, motivation, and learning [15]. In behavioral testing, DA is particularly involved in voluntary movement. There are four projection pathways in the central DA nervous system, among which the substantia nigra-striatal pathway plays an important role in motor regulation. In general, enhanced DA release in the striatum increases spontaneous locomotor activity, while DA suppression decreases this activity [45]. In the present study, a decrease in DA concentration in the striatum was expected because a decrease in spontaneous locomotion was observed in the IMI-exposed group in the behavioral test. However, there was no significant change in DA concentration in the striatum of the exposed group. A possible reason for this finding was the large variability in the control group. The Bartlett test for DA in the striatum was used to determine equivariance, and the results showed that the variances were different between groups. There was convergence of variance in the high-dose group, which may be due to neural disturbance caused by IMI administration, but the reasons are unknown. Note that the decrease in serotonin and histamine levels in the striatum was not dose-dependent. There are 2 possible reasons, which are as follows: (1) dose-dependent changes in the balance between secretion and synthesis due to feedback control and receptor downregulation; (2) secondary changes in MA levels due to other non-dose-dependent changes induced by IMI.

Acute exposure to a high dose of nicotine has been reported to decrease spontaneous locomotor activity in mice and rats [46,47]. Additionally, nAChRs are desensitized in the presence of nicotine, which is an agonist [48]. The release of DA in the striatum involves nAChRs being expressed on the dopaminergic nerves [49]. Furthermore, a previous study by Kimura-Kuroda et al. reported that both IMI and nicotine exhibit agonist activity toward mammalian nAChRs in vitro [50]. In other words, IMI may act as an agonist and desensitize nAChRs, or act as a partial agonist, thereby suppressing the release of DA into the synaptic cleft and reducing spontaneous movements. However, since the decrease in spontaneous locomotion in this study was only observed in the high-dose group, we cannot exclude the possibility that this effect is secondary to other stresses caused by exposure to high levels of NN. Although CLO, another NN, has been reported to induce DA release in the striatum of rats [14], it has not been reported to increase activity in behavioral tests and, in contrast, has been reported to decrease spontaneous locomotion in mice [18]. There are also in vitro studies that reported that CLO enhanced ACh-induced currents, while IMI suppressed them [51]. Although a simple comparison cannot be made because of differences in dosing methods and animal species, the differences in changes to DA and behavior between CLO and IMI may be due to differences in affinity and agonist activity for nAChRs.

Interestingly, DA and 3-MT were significantly decreased in the olfactory bulb in the IMI-exposed group. There is a population of approximately 10% DA-activating interneurons within the glomerular layer of the mammalian olfactory bulb [52]. In Parkinson’s disease, which is caused by the loss of DA-activating nerves in the substantia nigra-striatal pathway and the depletion of DA in the striatum, olfactory disturbances are often observed [53]. The role of DA in olfaction is complex, but it has been reported that blocking dopaminergic nerves projecting from the substantia nigra to the olfactory bulb causes olfactory dysfunction [54]. This indicates that changes in DA concentration in the olfactory bulb may cause olfactory dysfunction, and that IMI may affect olfaction by acting on the olfactory bulb of mice.

Histamine levels were significantly decreased in the striatum, cerebellum, and olfactory bulb of the exposed group in the present study. Histamine is synthesized in the hypothalamic raphe nucleus and is projected to most areas of the brain, where it plays a role in arousal, cognition, and feeding behavior in the central nervous system. Although the relationship between NNs and the decreased spontaneous movements observed in behavioral tests is unknown, to date there have been no reports of NN affecting histamine, and to the best of our knowledge, this is the first time that NN has been shown to affect histamine levels. Furthermore, there was a significant decrease in plasma histamine levels in the exposed group in this study. Histamine in the blood is mainly stored in mast cells and basophils, and its release is known to induce inflammation and allergic reactions. There are reports of increased blood histamine levels in Alzheimer’s disease patients and decreased blood histamine levels in schizophrenia patients [55,56]. This suggests that histamine in the blood not only acts as an inflammatory mediator but is also a biomarker for central nervous system diseases. In the present study, histamine levels were also decreased in both the brain and the plasma, suggesting that histamine changes in the central nervous system are reflected in the blood. In the future, histamine and other blood monoamines could be used as simple biomarkers in neurotoxicity studies.

## 5. Conclusions

Monoamine neurotransmitters such as DA and serotonin regulate brain functions such as behavior, memory, and learning, and their abnormalities are thought to be involved in the development of neurological disorders such as depression and Parkinson’s disease. NNs are pesticides widely used worldwide because of their high selective toxicity to insects and their high safety profile. However, effects of NN on mammals have been reported in recent years, and symptoms suspected to be related to monoamines, such as altered spontaneous movements and induction of anxiety-like behavior in particular, have been observed. Despite these observations, the effects of NN on monoamines have not been clarified. Although mass spectrometry is reportedly useful for the measurement of monoamines, a highly sensitive analytical method has not been fully established because of the low ionization efficiency of these neurotransmitters. In this study, we improved the ionization efficiency by using DPP, and clarified the effect of NN administration on the distribution and concentration of monoamines in the brain.

The results of behavioral tests showed that IMI administration caused a decrease in spontaneous locomotion in mice. Furthermore, we measured the concentration of monoamines in various parts of the brain and a decreasing but not significant trend of DA was observed in the striatum, which is believed to be involved in spontaneous movements. However, decreases in 3-MT and DA in the olfactory bulb, and decreases in serotonin and histamine in the striatum were detected. In particular, changes in DA in the olfactory bulb occurred at the NOAEL dose, indicating that monoamine disruption is caused by low level of IMI. Furthermore, the measurement of blood monoamine levels showed a significant decrease in histamine in the exposed group. This paralleled the brain levels, indicating that blood histamine levels may be a biomarker reflecting the effects of CNS diseases [57,58].

Although we were unable to identify the cause of the effect of IMI on spontaneous locomotion, we were able to show, for the first time by LC/MS quantification using DPP derivatization, that NN alters monoamine concentrations in various parts of the brain, even at a low-dose exposure.

## Figures and Tables

**Table 1:** The weight of each part of brain.

Brain Region	Control [mg]	Low-Dose [mg]	High-Dose [mg]
Striatum	3.30 ± 0.20	3.09 ± 0.21	3.84 ± 0.23
Hippocampus	6.54 ± 0.55	5.84 ± 0.47	7.25 ± 0.46
Cortex	54.29 ± 5.18	62.36 ± 5.89	69.30 ± 1.04
Cerebellum	89.14 ± 5.27	95.61 ± 3.46	88.61 ± 2.08
Brain stem	65.90 ± 3.12	68.86 ± 1.46	68.70 ± 1.70
Olfactory bulb	20.15 ± 1.04	18.46 ± 1.68	18.69 ± 1.15

**Table 2:** MRM parameters for determination of monoamines.

Compound	RT [min]	MRM Transition [*m/z*]		CE [V]
		Precursor Ion	Product Ion	
DPP-3-MT	3.030	383.1	151.2	24
			91.0	60
DPP-Dopamine	2.860	369.0	233.3	24
			233.2	20
DPP-Histamine	2.060	327.0	95.1	32
			233.4	28
			68.1	72
DPP-Serotonin	2.930	392.0	160.0	32
			232.3	20
DPP-3-MT-d4	3.030	387.3	156.2	24
			96.0	68
DPP-Dopamine-d4	2.860	372.1	232.1	28
			140.9	28
			94.5	56
DPP-Histamine-d4	2.060	330.0	232.2	24
			99.1	36
DPP-Serotonin-d4	2.930	395.0	164.2	24
			232.1	16

Target ion is the product ion listed in the top column of each compound. Other product ions are used as qualifier ions.

**Table 3:** IDL and MDL for monoamines.

Compound	IDL [ppt]	MDL [ppt]
3-MT	0.90	2.08
Dopamine	4.48	24.9
Histamine	3.24	9.82
Serotonin	0.91	1.51

IDL, Instrument detection limit; MDL, Method detection limit; 3-MT, 3-methoxytyramine HCl.

**Table 4:** The concentration of monoamines in different parts of brain.

Brain Region	Control	Low-Dose	High-Dose
3-MT [ng/g]			
Striatum	747.3 ± 88.0	807.4 ± 137.1	595.6 ± 39.9
Hippocampus	6.1 ± 1.2	5.0 ± 0.6	4.9 ± 1.0
Cortex	66.7 ± 15.6	69.0 ± 15.8	56.5 ± 5.2 ☨
Cerebellum	3.1 ± 0.2	3.7 ± 0.1	4.4 ± 0.45 *
Brain stem	13.0 ± 0.9	11.9 ± 0.6	13.9 ± 1.2
Olfactory bulb	72.4 ± 5.1	62.4 ± 3.3	51.8 ± 1.4 **☨
Dopamine [ng/g]			
Striatum	9747.0 ± 1580.2	9364.5 ± 1458.3	8283.8 ± 515.5 ☨
Hippocampus	67.9 ± 8.7	46.3 ± 8.8	43.0 ± 9.6
Cortex	418.0 ± 103.4	457.7 ± 116.7	294.3 ± 34.7 ☨
Cerebellum	20.2 ± 1.6	16.7 ± 0.7	13.7 ± 0.7 ***☨
Brain stem	123.0 ± 7.3	106.6 ± 7.3	116.9 ± 8.0
Olfactory bulb	407.8 ± 20.0	331.9 ± 16.7 **	304.8 ± 10.1 ***
Histamine [ng/g]			
Striatum	452.8 ± 28.3	133.7 ± 28.5 ***	198.1 ± 12.2 ***
Hippocampus	53.3 ± 6.8	48.3 ± 4.8	39.6 ± 5.3
Cortex	64.1 ± 4.9	57.9 ± 4.1	55.9 ± 4.5
Cerebellum	60.9 ± 4.0	54.7 ± 3.2	44.9 ± 4.0 *
Brain stem	65.3 ± 10.4	63.5 ± 4.8	60.8 ± 5.6
Olfactory bulb	155.9 ± 16.2	112.9± 8.6 *	115.3 ± 7.6 *
Serotonin [ng/g]			
Striatum	438.0 ± 44.8	193.3 ± 33.7 ***	320.2 ± 14.8 *☨
Hippocampus	704.0 ± 78.3	800.0 ± 51.7	651.3 ± 55.8
Cortex	546.6 ± 40.0	565.6 ± 41.9	481.3 ± 24.8
Cerebellum	370.1 ± 36.8	404.4 ± 30.5	260.8 ± 24.5
Brain stem	1348.0 ± 56.8	1407.9 ± 49.9	1288.4 ± 69.4
Olfactory bulb	666.8 ± 26.8	662.2 ± 43.3	616.9 ± 38.1

Values represent the mean ± SE of 8 animals in each group. * *p* < 0.05, ** *p* < 0.01, *** *p* < 0.001, Dunnett’s test; ☨ *p* < 0.025, Bartlett test.

**Table 5:** The concentration of monoamines in the plasma.

[ng/mL]	Control	Low-Dose	High-Dose
3-MT	0.91 ± 0.13	0.99 ± 0.078	1.05 ± 0.13
Dopamine	N.D.	N.D.	N.D.
Histamine	131 ± 32.78	58.90 ± 6.88 *	45.08 ± 3.43 **
Serotonin	1235 ± 261	1460 ± 580	1075 ± 159

N.D., Not detected. Values represent the mean ± SE of 8 animals in each group (ng/mL). * *p* < 0.05, ** *p* < 0.01, Dunnett’s test.

## Data Availability

The datasets generated and/or analyzed during the current study are available from the corresponding author upon reasonable request.

## Supplementary Materials

The following supporting information can be downloaded at: https://www.mdpi.com/article/10.3390/toxics10110696/s1, Table S1: The accuracy, precision and linearity of monoamine analytical method.
