# Supplementary material for: Detection of Changes in Monoamine Neurotransmitters by the Neonicotinoid Pesticide Imidacloprid Using Mass Spectrometry
Source: Toxics. 2022 Nov 17;10(11):696. doi: 10.3390/toxics10110696 (PMC9695199; doi:10.3390/toxics10110696)
Supplement: Supplementary file 1 [file toxics-10-00696-s001.zip › toxics-1997435-supplementary.pdf]

Table S1. The accuracy, precision and linarity of monoamine analytical method**.**

**Compound**	**Accuracy [%]**			**Precision [CV%]**	**Linarity [R^2]**
	0.2 ppb	2 ppb	20 ppb		
3-MT	99.6±8.3	98.4±6.5	104.4±5.5	64.8	0.9998
Dopamine	135.8±8.2	100.9±3.1	100.2±3.5	31.3	0.9998
Histamine	269.4±6.7	90.1±4.1	100.4±0.4	26.9	0.9995
Serotonin	155.4±28.0	94.7±3.5	98.9±2.2	11.6	0.9999

CV, Coefficient of variation

These values were calculated from the measurement results and calibration curve used to calculate the MDL.

Accuracy was represented as the mean ± SE, and linearity was shown as R^2 on the calibration curve.
